# A Novel Method of UAV-Assisted Trajectory Localization for Forestry Environments

**DOI:** 10.3390/s24113398

**Published:** 2024-05-25

**Authors:** Jian Huang, Xiansheng Guo

**Affiliations:** Department of Electronic Engineering, University of Electronic Science and Technology of China, Chengdu 611731, China; huangjian@funmitech.com

**Keywords:** multi-agent deep reinforcement learning, least squares, trajectory localization, equipment heterogeneity

## Abstract

Global positioning systems often fall short in dense forest environments, leading to increasing demand for innovative localization methods. Notably, existing methods suffer from the following limitations: (1) traditional localization frameworks necessitate several fixed anchors to estimate the locations of targets, which is difficult to satisfy in complex and uncertain forestry environments; (2) the uncertain environment severely decreases the quality of signal measurements and thus the localization accuracy. To cope with these limitations, this paper proposes a new method of trajectory localization for forestry environments with the assistance of UAVs. Based on the multi-agent DRL technique, the topology of UAVs is optimized in real-time to cater for high-accuracy target localization. Then, with the aid of RSS measurements from UAVs to the target, the least squares algorithm is used to estimate the location, which is more flexible and reliable than existing localization systems. Furthermore, a shared replay memory is incorporated into the proposed multi-agent DRL system, which can effectively enhance learning performance and efficiency. Simulation results show that the proposed method can obtain a flexible and high-accuracy localization system with the aid of UAVs, which exhibits better robustness against high-dimensional heterogeneous data and is suitable for forestry environments.

## 1. Introduction

Forest fires can cause severe damage to animal and plant resources as well as the ecological environment, leading to soil erosion, significant economic losses, and even casualties. During the firefighting process, in order to achieve the most optimized firefighting strategy and plan the optimal escape routes, the location information of the firefighters is particularly crucial. Normally, a global navigation satellite system (GNSS) can provide satisfactory localization and navigation performance. However, in the forestry environment, the GNSS signals are highly vulnerable to environmental uncertainties, rendering the localization result unreliable, especially under emergency security scenarios [1,2].

In recent years, machine learning (ML) algorithms have been widely used [3,4], and reinforcement learning (RL), as a major branch of ML, has also attracted much attention. The rise of RL has brought great progress in the field of localization [4]. Dou et al. [5] proposed a hierarchical framework for two-dimensional localization in which DRL was used to continuously move and shrink the two-dimensional plane window until the target accuracy was achieved. This localization framework requires no prior knowledge of plane plans in the environment. Moreover, Dou et al. [6] extend the two-dimensional localization scheme into a hierarchical framework for 3D localization, which can provide more information and functionality in the IoT era. By constantly moving and shrinking the cube form, DRL is used to continuously divide the search space, starting from the whole building until the preset target position is reached. Moreover, Mohammadi et al. [7] proposed a semi-supervised Deep Reinforcement Learning (DRL) model in which the agent moves step by step in a grid area according to the designed actions until the target is accurately located. Similarly, Li et al. [8] proposed a localization model utilizing a novel reward function based on near-field conditions and the location of the wireless gateway, which is the first DRL localization approach without a site-survey process. The above methods are almost based on RSS technology, which is inaccurate in complex environments. In order to enhance localization accuracy using RSS, some researchers consider that RL can be assisted by Unmanned Aerial Vehicles (UAVs), which can measure the RSS of objects from multiple different angles. It also has a higher Line-of-Sight (LoS) probability. Testi et al. [9] used RSS as a localization signal source and the RL algorithm to find the best spatial configuration of UAVs to locate the target in an unknown environment. Afifi et al. [10] proposed a geometry-based localization algorithm based on 5G RSS measurements from four base stations for 3D UAV localization, which has the advantage of providing practical real-time calculation for localization problems compared with typical deep learning algorithms.

Existing UAV-assisted localization frameworks fall into the category of trilateration localization frameworks, the accuracy of which highly depends on the quality of signal measurements. Moreover, the RL-based UAV-assisted localization schemes suffer from the following issues: Traditional Q-learning algorithms store state-action data in Q-tables, which can only cope with low-dimensional state RL problems. Moreover, traditional RL algorithms are typically designed for single-agent systems, while in actual UAV-assisted localization problems, multiple-UAV formulations can expand the exploring space and promote multi-agent perception capability.

To address the above problems, we propose a multi-agent deep reinforcement learning (multi-agent DRL)-based trajectory localization framework for UAVs. Firstly, a least squares (LS) algorithm is employed to estimate the location of targets based on RSS measurements. As proved in the literatures [11,12], the localization accuracy of the LS estimator can approximate the Cramér–Rao lower bound. Then, we utilize the multi-agent deep Q-network (multi-agent DQN) scheme to navigate the UAVs to form a better topology, which can perform better localization for the target by autonomously getting rid of channel uncertainties. In the process of DQN training, we employ the labels of trajectory data to set reward functions and iteratively update the network parameters using gradient decent methods until convergence. In the simulation settings, we model different UAVs as agents with different environments and noise parameters, which corresponds with the device heterogeneity issues in RSS localization. The main contributions of this paper are summarized as follows:

- For different existing localization systems [2,3,13,14,15,16,17], the proposed multi-agent DRL-based trajectory localization framework employs easy-deployed UAVs as the signal anchors and eliminates the requirements for several pre-deployed anchors with fixed locations, which is more feasible for the complicated and changeable forestry environments, especially in the emergency rescue process.
- To cope with the environmental uncertainty and heterogeneity among agents, which severely degrade the localization performance, the proposed trajectory localization method utilizes the multi-agent DRL technique to automatically navigate the UAVs to form an optimal topology in real-time, allowing higher-accuracy localization for the targets.
- Moreover, by developing a shared replay memory for multi-agent interactions, the complementary information among agents can be utilized to enhance learning efficiency and performance, which contributes to superior and robust localization performance.

## 2. Preliminaries and Problem Formulation

Assume that the location of the target to be positioned is $x={\left[x,y\right]}^{T}$, the location of the i UAV equipped with sensors is $x_{l}={\left[x_{l},y_{l}\right]}^{T}$, l=1,2,…,L. L≥3 represents the number of sensors. $x_{l}$ is known as prior information. The measurement information of these drones and targets, as well as the position information of these sensors, is expected to be used to work out the actual location of the target.

The received signal strength (RSS) is the average received power, which is widely employed in many fields [18,19] by virtue of its easy availability. It is generally assumed that the signal propagation follows an exponentially decayed path loss model, which is a function of the transmit-receive distance, path loss factor, and transmitted power. RSS localization has a lower implementation cost than TOA/TDOA localization because it does not require time synchronization between transmit, receive, and receiver. As long as the distance between the transmitting and receiving base stations is estimated, the position can be solved using the trilateral localization of the TOA.

Assume that the transmitting power is $P_{t}$, the receiving power of the ith UAV $P_{r,i}$ can be expressed as follows:

(1) $$P_{r,i}=K_{i}P_{t}{\left\|x-x_{i}\right\|}_{2}^{-\alpha },i=1,2,\cdots ,M,$$

where $K_{i}$ is the receiving-transmitting gain depending on the height, gain of the antenna, and α∈[2,5] is the path loss element. Empirically, α=2 in a free-space propagation environment. Equation (1) can be rewritten in the following logarithm form:

(2) $$\ln\left(P_{r,i}\right)=\ln\left(K_{i}\right)+\ln\left(P_{t}\right)-\alpha \ln\left(d_{i}\right)+n_{RSS,i},$$

where $n_{RSS,i}$ is a zero-mean Gaussian noise and $\sigma_{RSS,i}^{2}$ is the variance. $d_{i}$ denotes the distance between the target and the ith sensor, which can be calculated as follows:

(3) $$d_{l}=\sqrt{{\left(x-x_{l}\right)}^{2}+{\left(y-y_{l}\right)}^{2}}.$$

With the definition of RSS as follows: $r_{RSS,i}=\ln\left(P_{r,i}\right)-\ln\left(K_{i}\right)-\ln\left(P_{t}\right)$, Equation (2) can be expressed as follows:

(4) $$r_{RSS,i}=-\alpha \ln\left(d_{i}\right)+n_{RSS,i},i=1,2,\cdots ,L.$$

For notation conciseness, it can be further written in the following vector form:

(5) $$r_{RSS}=f_{RSS}\left(x\right)+n_{RSS},$$

where

(6) $$r_{RSS}={\left[r_{RSS,1},r_{RSS,2},\cdots ,r_{RSS,L}\right]}^{T},$$

(7) $$n_{RSS}={\left[n_{RSS,1},n_{RSS,2},\cdots ,n_{RSS,L}\right]}^{T},$$

(8) $$f_{RSS}\left(x\right)=p=-\alpha \left[\begin{array}{c} \ln\left(\sqrt{{\left(x-x_{1}\right)}^{2}+{\left(y-y_{1}\right)}^{2}}\right)\\ \ln\left(\sqrt{{\left(x-x_{2}\right)}^{2}+{\left(y-y_{2}\right)}^{2}}\right)\\ \vdots \\ \ln\left(\sqrt{{\left(x-x_{L}\right)}^{2}+{\left(y-y_{L}\right)}^{2}}\right) \end{array}\right].$$

**Figure 1:**
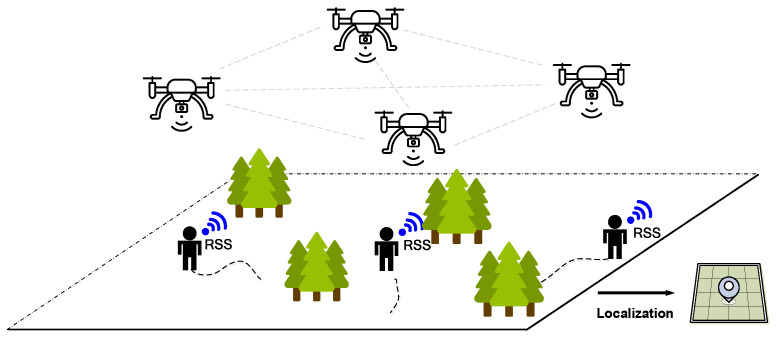
Main architecture of the proposed UAV-assisted positioning method.

The goal of trajectory localization is to estimate the location of the target in real time based on the RSS measurements. Traditional localization schemes [11,20,21] utilize linear least squares (LLS), weighted linear least squares (WLLS), or other regression methods to estimate $x={\left[x,y\right]}^{T}$. The position of the sensor is fixed and known. However, this assumption is difficult to meet in the post-disaster rescue environment because the fire may cause drastic environmental changes at any time, which will seriously interfere with the reliability of communication and sensing equipment. Therefore, the use of pre-deployed sensor networks to provide location services is not reliable. Moreover, the topology of UAVs severely restricts the localization accuracy of targets. For example, if the RSS measurements from some UAVs are blocked by barriers like trees or walls, the localization performance using LLS may be terrible. In order to solve these problems, this paper adopts a UAV equipped with RSS sensors, which can effectively build a flexible sensor network and provide an observation platform, allowing for the formation of the optimal UAV topology in pursuit of high-precision localization of targets. The goal of this paper is to predict the movement trajectory of users in real time based on the RSS data collected by UAVs, so as to ensure the safety of personnel and assist in the subsequent rescue work. The main architecture of our localization system is given in Figure 1.

## 3. Proposed Multi-Agent DQN-Based Method

In this section, we elaborate on the UAV-assisted positioning procedure. Firstly, in order to find the optimal UAV topology for accurate localization, we model the positioning framework as a Markov decision process. A MDP system consists of four key components: state space S, action space A, reward function r∈R, and state transition probability $p(s_{t+1}|s_{t},a_{t})$, where $s_{t}\in S$ and $a_{t}\in A$ represent the state and action of the agent at time t, respectively. The objective of MDP is to find an optimal policy to maximize the expected accumulated reward $R_{t}=\sum_{i=1}^{\infty }\left(\gamma_{i}r_{t+i}\right)$, where $r_{t+i}$ is the reward at time t+i and $\gamma_{i}\in [0,1]$ is the discount factor. The MDP in this paper is modeled as follows:

**State space**: In the proposed MDP model, the state is composed of four parts: (1) L agents with known coordinates $x_{1},x_{2},\ldots ,x_{L}$; (2) n history actions (each action is encoded by a one-hot code, where the encoded bit is the dimension of the action); (3) the RSS sequences obtained by UAVs from targets along a trajectory; (4) a mark that judges whether the target is inside the localization region.

**Action space**: We split the localization region into equally spaced grids, and the action space of each agent consists of nine actions, i.e., staying at the same grid and moving toward north, south, west, east, northwest, northeast, southwest, and southeast for a grid. In each step of movement, the agent takes an action from the action space.

**Reward function**: The reward function is designed based on the localization accuracy, which is measured by the estimated error $d=||x-\hat{x}||$ between the estimated location and actual location, where $\hat{x}$ denotes the estimated location using LLS\WLS methods. If the estimated error is undesirably larger than a predefined threshold, the current topology of UAVs is not beneficial for accurate localization, and a penalty should be given. In contrast, a relatively small, estimated error indicates that the localization performance is acceptable. Hence, a reward should be given, and the smaller d is, the bigger the reward should be set. On the other hand, based on the near-field condition, i.e., a strong RSS value can ensure a short distance between agents and the target, we give the agent a reward or penalty if and only if the average distance between agents and the target is smaller than a pre-defined threshold $d_{0}$. If the estimated location is within the threshold scope, we give the agent a positive reward equal to the inverse of the reciprocal of the estimated error. In contrast, we give the agent a penalty if the estimated location is outside the threshold, which is the negative value of the estimated error. The agent receives no reward or penalty if the average distance between agents and the target is greater than $d_{0}$. The reward function is computed as follows:

(9) $$r=\{\begin{array}{l} \frac{1}{d},\;\;d_{n}\leq d_{0}\;\&\;d<d_{th}\\ -d,\;d_{n}\leq d_{0}\;\&\;d\geq d_{th}\\ 0,\;\;\;d_{n}>d \end{array},$$

where $d_{n}$ is the average distance between 3 agents and the target, and $d_{th}$ denotes the distance threshold for location estimates.

**Figure 2:**
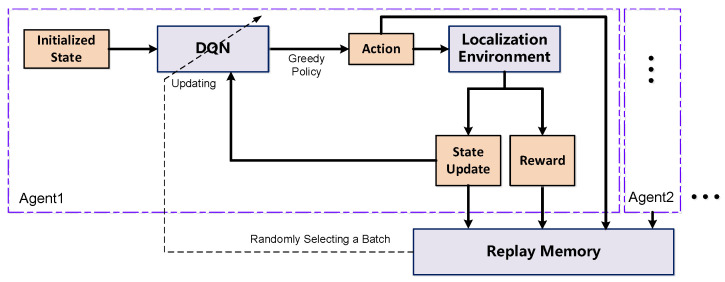
Architecture of the multi-agent DQN.

The positioning framework proposed in this paper assumes communication between the UAV and the target, as shown in Figure 2. The model uses the least squares model to estimate the location of the target and then uses the multi-agent DRL algorithm to navigate the UAVs to autonomously form the optimal topology. The RSS in the environment is utilized when estimating the location of the target and the UAV, and their label location information is used in the calculation of the reward function in the multi-agent DRL algorithm in the training process. In the process of algorithm execution, it is assumed that the target moves forward first; the UAV can measure the RSS of the target at the moment by itself and estimate the target position according to the LLS/WLLS algorithm. At time t−1, each UAV moves forward by taking an action based on the trained DQN in order to find a better placement for target localization.

As shown in Figure 2, this paper adopts the Deep Q Network (DQN) model to solve the above problems. DQN is a reinforcement learning optimization method in Q-learning. The goal of Q-learning is to solve the following functions:

(10) $$Q:s_{t}\to Q\left(s_{t},a_{t};\theta \right),$$

where θ is the action-value model parameter, which is used to map the state of the input to the decision of the output. This function can be used to calculate the cumulative expected reward for taking action $a_{t}$ in state $s_{t}$. With the aid of Equation (10), we can obtain the maximization strategy in the current state:

(11) $$\pi (s)=\underset{a}{\mathrm{argmax}}Q(s,a).$$

In traditional Q-learning, the Q function can be calculated by the Q matrix method, but in this task, due to the uncertainty and complexity of the localization task, it is difficult to use limited state space to model this continuous problem. Therefore, deep networks are used in this paper to approximate the function in this continuous space, namely DQN.

In the training of DQN, the input is the current state, and the output includes the reward values for each possible action and the state for the next step. We save this result in an experience replay memory D. At each step, a small batch of data is randomly drawn from D to calculate the loss function and then update the parameters in the DQN. Generate an experience replay memory of capacity $N_{ep}$ when initializing the model, and then store each experience sample. When the number of experiences in the playback pool reaches the threshold $N_{st}$, a batch of $N_{mb}$ samples is randomly selected to train the network. At the same time, the epsilon-greedy policy is used to select the action in the current state. This strategy balances knowledge based on the DQN model (development) and, by trying out new behaviors (exploration), maximizing the reward for acquiring new knowledge. The explored factor ϵ decays linearly from an initial value $\epsilon_{0}$ until a minimum value $\epsilon_{f}$ is reached. For each experience sample, the following loss function is calculated as follows:

(12) $$L(\theta )=E\left[{\left(y_{j}-Q\left(\phi (s),a;\theta_{j}\right)\right)}^{2}\right],$$

where E[·] represents the expected value for ⋅, and $y_{j}$ is the target value calculated by the following:

(13) $$y_{j}=r_{j+1}+\gamma \max_{a}\hat{Q}\left(\phi \left(s_{j+1},a;\theta^{-}\right)\right).$$

Based on this loss function, DQN is trained using the stochastic gradient descent (SGD) method. We summarize the training process of the proposed multi-agent DQN-based trajectory localization framework in Algorithm 1.
**Algorithm 1.** Training process for the UAV-assisted trajectory localization framework
**Input:** The RSS sequence, the trajectory of the UAVs, and the trajectory for the target.
**Output:** DQN parameters.
1: Initialize the model parameters, environment, space, and experience replay memory.
2: **for** episode in 1 to *M*
**do**:
3:      **for** each trajectory **do**:
4:           Utilizing the LLS/WLLS scheme to estimate the initial location for the target
5:           **for** *t* in 1 to *T_max_* **do**:
6:                for agent in 1 to L do:
7:                     Select an action using epsilon-greedy policy
8:                     Execute the action and obtain reward *r*, next state *s*′
9:                     Navigate the UAV itself to next placement
10:                   Estimate the location for the target in time *t* using LLS/WLLS 
11:                   Store the experience into the replay memory
12:                   Randomly select a batch from the replay memory
13:                   Using Equations (12) and (13) to update DQN
14:              **end for**
15:         **end for**
16:    **end for**
17: **end for**

After training the DQN, the UAVs can autonomously navigate themselves to the optimal placement for target localization, and then, by using LLS/WLLS methods, an accurate and robust localization result can be obtained.

In order to comprehensively evaluate the proposed algorithm, we further analyze its complexity subsequently. Notably, we mainly focus on the computational complexity of the online UAV-assisted localization process rather than the training process that typically takes place on computation-intensive central servers or simulation platforms. Firstly, we assume that the DQN, our algorithm, is composed of basic, fully connected layers, such that the computational complexity of Algorithm 1 mainly depends on the size of the neural network and the learning rate of the agent. For each execution time, the complexities of operating the DQN are $O(N_{S}N_{H}+N_{H}N_{A})$, with $N_{S}$, $N_{H}$, and $N_{A}$ denoting the dimensions of the state space, the hidden layer of the DQN, and the action space, respectively. For a system consisting of L agents, the policy is operated $L\times T_{max}$ times to optimize the Q value in Equation (13). After the UAVs form an optimal topology, LLS/WLLS methods will be executed to localize the target, which costs a complexity of $O(L^{3}+L^{2})$ for two-dimensional localization. Hence, the execution complexity of the proposed method is $O(LT_{max}N_{H}(N_{S}+N_{A})+L^{3})$. Note that in our work L=3, $T_{max}\leq 20$, $N_{A}=9$, and $N_{S}\leq 20$, which makes the executing complexity acceptable for modern digital processers.

## 4. Simulation Results and Analyses

### 4.1. Dataset Description

The data in this simulation mainly consist of trajectory data and RSS data. The trajectory data further include target trajectory data (used for the computation of the reward function and the evaluation of localization performance) and the agent’s trajectory data (used for the computation of the reward function). The RSS data contain the RSS data received by the agent at moment t and the location of the target at t−1 moment. The trajectory data meet the condition that UAVs do not collide with each other and their respective distances from the target do not exceed a certain limit $d_{0}$.

With the aid of path loss models [22], the RSS value $r_{l}$ from the lth sensor is generated as follows:

(14) $$r_{l}=-\alpha \ln(d_{l})+n_{l},\;\;\;l=1,2,3,$$

where α is the path loss exponent (PLE), which depends on the multi-path properties in a certain environment and ranges from 1 to 5. Empirically, the PLE satisfies 2≤α≤5 and 1≤α<2 under outdoor and indoor scenarios, respectively. In the free space, we set α=2. $d_{l}$ denotes the Euclidean distance between the lth sensor and the signal source. $n_{l}$ is a random variable describing the path loss and can be expressed as follows:

(15) $$n_{l}=0.1\ln(10)w_{l}.$$

Without loss of generality, $w_{l}$ can be modeled as a zero-means Gaussian variable with a known variance modeled as follows:

(16) $$\lambda_{l}^{2}=0.01{\left(\ln(10)\right)}^{2}\sigma_{l}^{2},\;\;l=1,2,\ldots ,L,$$

where $\sigma_{l}^{2}$ is the variance value and is known to us. Typically, we assume that small-scale fading can be ignored^.^ Hence, we set α=2, σ=1, α=1.6, σ=6, and α=1.9, and σ=6 to simulate heterogeneous situations, which can be referred to in the literature [21].

### 4.2. Environmental Setting

The involved parameters in the training of the proposed multi-agent DLR algorithm are listed in Table 1.

### 4.3. Evaluation of Localization Performance

#### 4.3.1. Training Process

**Figure 3:**
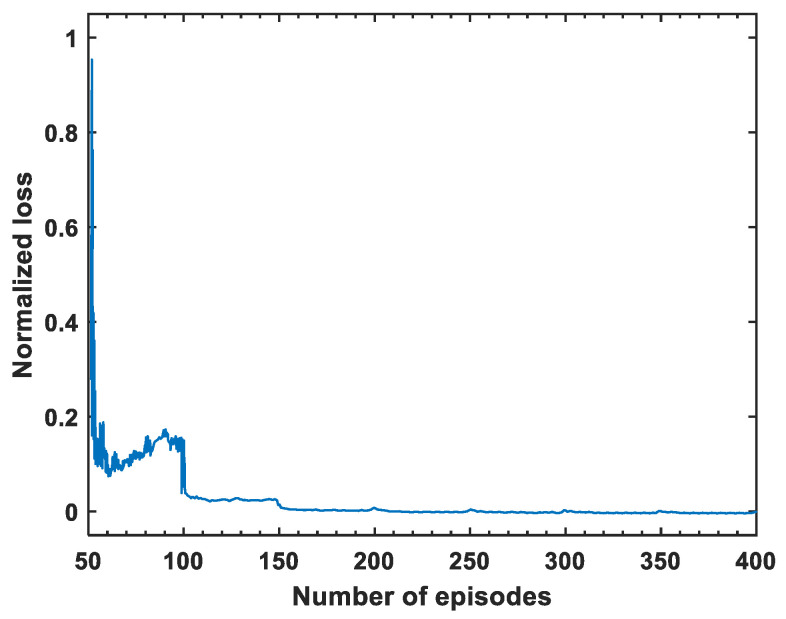
Loss convergence trend.

The normalized loss of multi-agent DQN in the training process is presented in Figure 3, where the loss monotonically decreases and converges to zero, validating the convergence of DQN.

**Figure 4:**
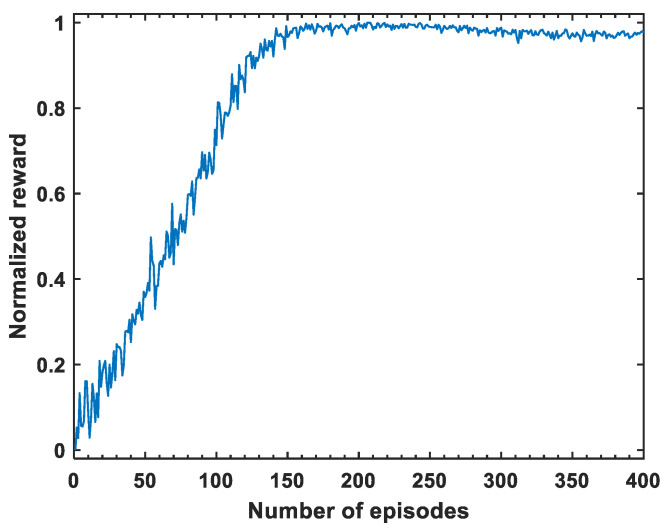
Changing trend of normalized reward value during training iteration.

The trend in the normalized cumulative reward value during the training process of the model is shown in Figure 4. The normalized cumulative reward value fluctuates significantly. However, as can be seen from this figure, it generally shows an increasing trend, which is consistent with the objective of DRL training. And finally, the reward converges after about 150 episodes, which lays a solid foundation for the subsequent localization process.

#### 4.3.2. Testing Process

In the testing process, we generate simulation data under different noise and environmental conditions. We utilize 100 traces, each of which consists of 100 steps for simulation, and 3 performance indices to evaluate the trajectory estimation performance, i.e., average localization error (ALE), root mean square error (RMSE), and minimum localization error (MLE) [23], which are calculated as follows:

(17) $$ALE=\frac{\Sigma d_{i}}{n},RMSE=\sqrt{\frac{\Sigma d_{i}^{2}}{n}},MLE=\min d_{i},$$

where $d_{i}$ is the normed error between the ith estimated location and the ith actual location and n is the number of testing samples.

In the testing process, we chose LLS, WLLS, and improved LLS, as typical localization schemes assisted by our proposed multi-agent DRL technique, while trilateration [11] represents a classical geometry-based method without the proposed optimization procedures. The testing performance of different localization models under different environmental settings is presented in Table 2.

After analyzing the simulation results in Table 2, we can draw the following conclusions. This model is significantly affected by noise and environmental changes. In free space and under the condition that the distribution of the RSS data follows the standard normal distribution, the ALE, RMSE, and MLE of 100 trajectory data are commonly small using LLS, WLLS, or improved LLS algorithms compared to other conditions. Specifically, under these conditions, the errors for the multi-agent DQN algorithm, LLS, WLLS, and improved LLS algorithms are 3.439 m, 3.503 m, and 3.410 m, respectively, while the error for trilateration is 4.419 m. With the standard deviation of RSS data distribution being constant, the closer the environmental conditions are to free space, the better the performance of the algorithms.

**Figure 5:**
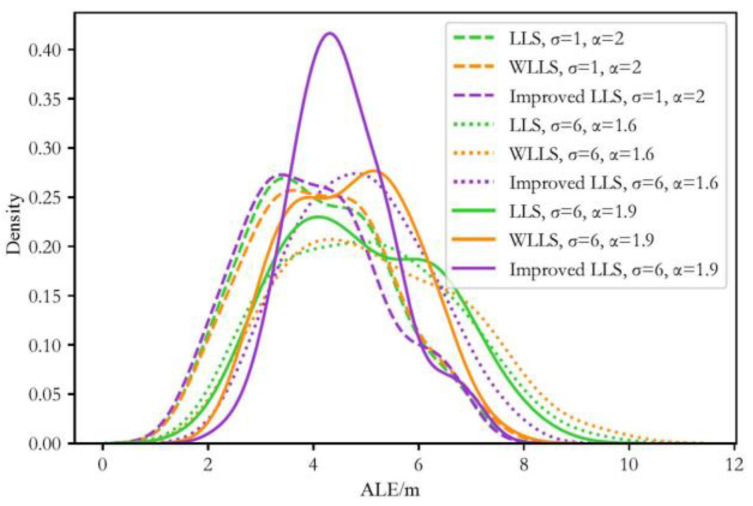
Kernel density distribution diagram of the average localization error of isomorphic data with different environments and noise.

To more intuitively compare the performance of the algorithm under different noise and environmental conditions, as well as with different LS-solving methods, we present the positioning errors of 100 test trajectories in the form of kernel density distribution across various dimensions. From Figure 5, we can clearly observe the distribution density of the model’s positioning errors across different noise and environmental conditions. Under the same noise and environmental conditions, a similar distribution of ALEs can be found, which conforms to the results in Table 2.

Note that in practical applications in the forestry environment, the data variation problem due to the forest density and terrain features is inevitable, which greatly affects the distribution of received RSS measurements. Hence, we subsequently evaluate the localization performance with heterogeneous data, i.e., simulation with different noise conditions and environmental conditions. Specifically, we divide the data into seven groups based on different values of α and σ, train corresponding models, and test the results. Suppose UAV1 receives RSS simulation data from the target corresponding to conditions $\alpha_{1}$ and $\sigma_{1}$, UAV2 corresponds to $\alpha_{2}$ and $\sigma_{2}$, and UAV3 corresponds to $\alpha_{3}$ and $\sigma_{3}$.

**Figure 6:**
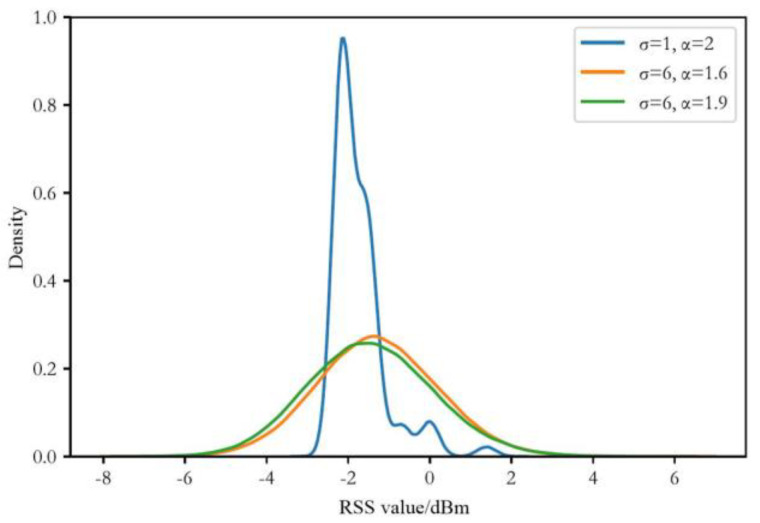
Kernel density distribution diagram of the RSS value with different noise and environmental conditions.

Firstly, we use the form of kernel density to show the distribution differences of RSS data under different noise and environmental conditions, as shown in Figure 6. The first group of RSS (obtained under conditions σ=1 and α=2) is assumed to be obtained in free space, and its RSS value distribution is relatively concentrated compared to the other two groups, with smaller overall distribution differences. The other two groups of RSS, since they have the same standard deviation, both with σ=6, but slightly different α values, have similar kernel density distributions. The standard deviations of these two groups of RSS are larger than the first group, resulting in greater differences in RSS value distributions and less concentrated values. Therefore, we will combine these data sets with significant RSS value distribution differences to discuss the performance of the algorithm proposed in this paper under heterogeneous situations, corresponding to the common device heterogeneity problem in RSS localization. The localization error evaluations of heterogeneous data are shown in Table 3.

As can be seen from Table 3, the algorithm of the model shows different performance under various least squares (LS)-solving methods, as well as under different noise and environmental conditions. It can be concluded that different noise and environmental conditions are suitable for different LS-solving methods. The linear least squares (LLS) algorithm performs best in the third group of heterogeneous data, specifically when the noise and environmental condition parameters for UAV1 are set to $\alpha_{1}=2$ and $\sigma_{1}=1$, and those for UAV2 and UAV3 are set as $\alpha_{2}=1.6$ and $\sigma_{2}=6$. Under these conditions, the average positioning errors and RMSE for the multi-agent DQN and LLS algorithm are 4.611 m and 3.816 m, respectively. The weighted linear least squares (WLLS) algorithm performs best in the second group of heterogeneous data, specifically when the noise and environmental condition parameters for UAV1 and UAV2 are set to $\alpha_{1}=2$, $\sigma_{1}=1$, and those for UAV3 are set to $\alpha_{3}=1.9$, $\sigma_{3}=6$. Under these conditions, the average positioning errors and RMSE for the multi-agent DQN and WLLS algorithms are 3.990 m and 3.429 m, respectively. The improved LLS algorithm performs best in the first group of heterogeneous data, specifically when the noise and environmental condition parameters for UAV1 and UAV2 are set as $\alpha_{1}=2$ and $\sigma_{1}=1$, and those for UAV3 are set as $\alpha_{2}=1.6$ and $\sigma_{2}=6$. Under these conditions, the average positioning errors and RMSE for the multi-agent DQN and improved LLS algorithms are 4.343 m and 3.535 m, respectively.

**Figure 7:**
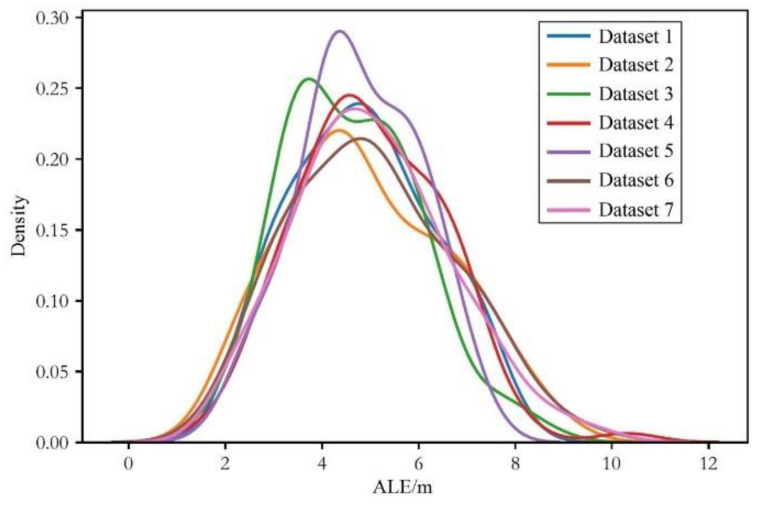
Kernel density distribution diagram of the average localization error of multi-agent DQN with the LLS algorithm with heterogeneous data.

**Figure 8:**
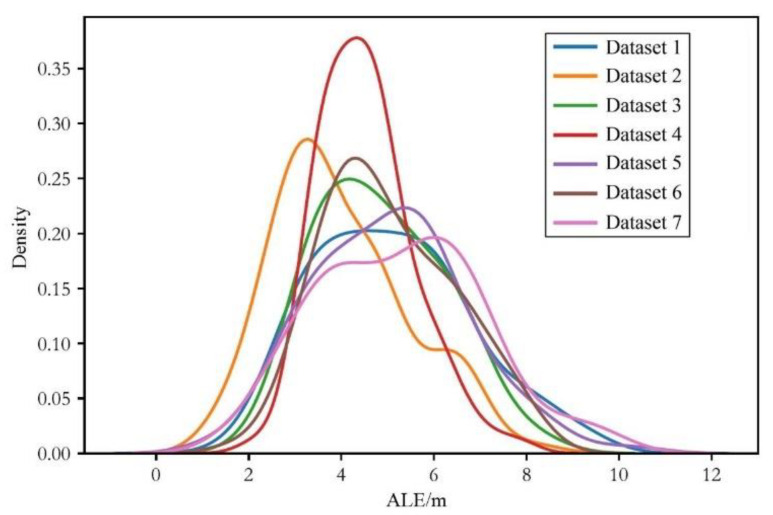
Kernel density distribution diagram of the average localization error of multi-agent DQN with the WLLS algorithm with heterogeneous data.

**Figure 9:**
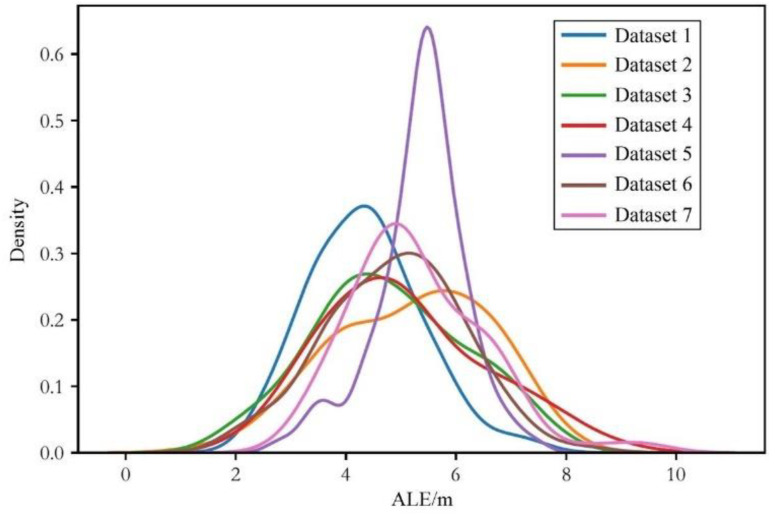
Kernel density distribution diagram of the average localization error of multi-agent DQN with the improved LLS algorithm with heterogeneous data.

The kernel density distributions of the average positioning error under different heterogeneous data are depicted in Figure 7, Figure 8 and Figure 9:

From the above figures, we can draw similar conclusions to Table 2. Furthermore, by analyzing the trend of the kernel density distribution curves in the figures, it can be observed that for different groups of heterogeneous data, the change in trend of the kernel density distribution curves of the LLS algorithm is not significant, while that of the WLLS algorithm shows greater variation, and the improved LLS algorithm exhibits the largest variation in trend. Therefore, it can be inferred that in the testing simulations with heterogeneous data, the LLS algorithm is the most stable in performance, followed by the WLLS algorithm, with the improved LLS algorithm being the least stable.

#### 4.3.3. Performance Comparison between the Proposed Scheme and the Traditional Trilateration Scheme

**Figure 10:**
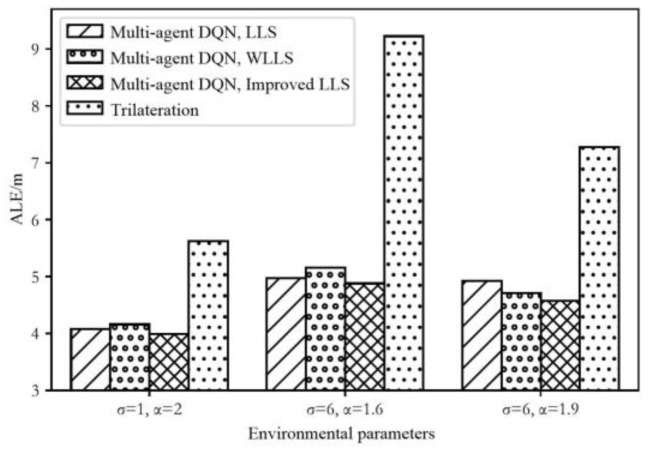
Average localization error of multi-agent DQN with different LS algorithms in different environments and noise conditions.

We use the trilateration method as a comparison to evaluate the average positioning error under different environmental and noise conditions. As illustrated in Figure 10, it is evident that the method proposed in this paper has significantly better adaptability to environmental changes compared to the trilateration method. In a quantitative analysis, the multi-agent DQN and three different solving methods of the LS algorithm, under simulation conditions with relatively high noise levels (where values are least close to free space in three sets of conditions), incur an average positioning error of 4.971 m, 5.159 m, and 4.879 m, respectively. In contrast, the average positioning error for the trilateration method is 9.225 m.

**Figure 11:**
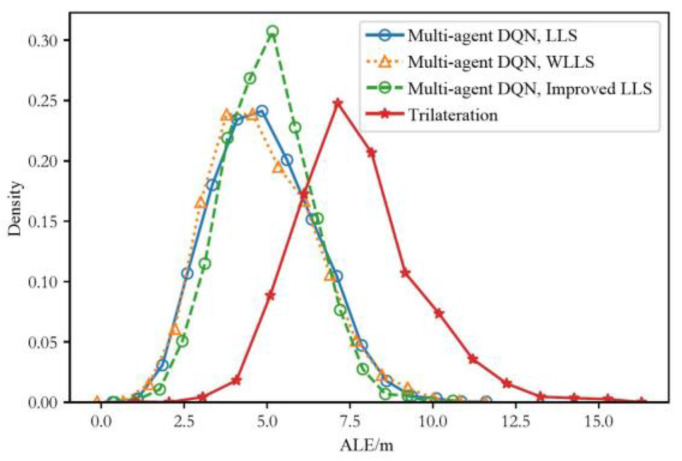
Kernel density distribution diagram of the average localization error of multi-agent DQN with different LS algorithms and heterogeneous data.

To more clearly demonstrate the advantages of the algorithm model proposed in this paper, we compared it with the trilateration method, evaluating the positioning error of the algorithm under device heterogeneity, as shown in Figure 11. The kernel density distribution of the average positioning error of the method proposed in this paper is significantly better than the traditional trilateration method under heterogeneous device conditions. As can be seen from the figure, both the trilateration method and the method proposed in this paper show a normal distribution of the average positioning error for each trajectory under heterogeneous data. The average positioning errors of the multi-agent DQN algorithm combined with LLS, WLLS, and improved LLS are mainly concentrated between 2.5 m and 7.5 m, with densities of 0.914, 0.907, and 0.954, respectively. In contrast, the average positioning error of the background technique mainly concentrates between 5 m and 10 m, with a density of 0.85. This indicates that under device heterogeneity, the average positioning error of the method proposed in this paper has a probability of over 90% being distributed between 2.5 m and 7.5 m, while the trilateration method has an 85% probability of being distributed between 5 m and 10 m. These results further validate the effectiveness and superiority of the proposed positioning scheme.

## 5. Conclusions

In this paper, we propose a UAV-assisted multi-agent DRL scheme to provide accurate location information for forestry environments, where GNSS signals are typically unstable or unavailable. Notably, the proposed positioning scheme avoids the need for fixed anchor points by using UAVs to provide ranging information, which is much more flexible and easier to deploy in forestry environments. Moreover, considering environmental uncertainty and equipment heterogeneity, we utilize the multi-agent DRL method to automatically navigate the UAVs to form an optimal topology for target localization and then estimate the target location with the aid of the LLS/WLLS algorithm. In addition, we incorporate a shared experience replay memory for multi-agent DRL to enhance the training performance and efficiency of different UAVs. Simulation results validate the effectiveness of the proposed UAV-assisted multi-agent DRL as an effective positioning solution for forestry environments.

## Figures and Tables

**Table 1:** Algorithm parameters.

Parameters			Values
Environmental parameters		Number of grid lines	10
		Number of grid volumes	10
		Grid size	1 m × 1 m
		Number of actions	9
		Number of agents	3
		Number of training traces	1000
		Number of testing traces	100
		Number of steps in each trace	100
Algorithm parameters	LS algorithm	Path loss exponent α	2/1.6/1.9
		Stand deviation σ	1/6
	multi-agent DRL algorithm	Size of replay memory	20,000
		Initial size of replay memory	2000
		Batch size for gradient decent	200
		Update frequency of the network	100
		Discount factor	0.99
		Learning rate	0.001
		Initial exploration rate	1
		Discount factor of exploration rate	0.002
		Final exploration rate	0.1

**Table 2:** Localization error evaluation for the simulation data under different noise and environmental conditions.

		ALE (m)	RMSE (m)	MLE (m)
α=2 σ=1	LLS	4.081	3.439	1.952
	WLLS	4.161	3.503	1.952
	Improved LLS	3.991	3.410	1.837
	Trilateration	5.623	4.419	1.495
α=1.6 σ=6	LLS	4.971	3.990	2.039
	WLLS	5.159	4.135	2.375
	Improved LLS	4.879	3.938	2.235
	Trilateration	9.225	16.384	5.918
α=1.9 σ=6	LLS	4.925	3.982	2.014
	WLLS	4.710	3.855	2.571
	Improved LLS	4.571	3.697	2.323
	Trilateration	7.274	8.919	4.860

**Table 3:** Localization error evaluation for simulated heterogeneous data.

		APE (m)	RMSE (m)	LPE (m)
$\alpha_{1}=2$, $\sigma_{1}=1$ $\alpha_{1}=2$, $\sigma_{1}=1$ $\alpha_{2}=1.6$, $\sigma_{2}=6$	LLS	4.840	3.889	2.191
	WLLS	5.084	4.070	1.870
	Improved LLS	4.343	3.535	2.326
	Trilateration	6.724	7.270	2.459
$\alpha_{1}=2$, $\sigma_{1}=1$ $\alpha_{1}=2$, $\sigma_{1}=1$ $\alpha_{3}=1.9$, $\sigma_{3}=6$	LLS	4.944	4.012	2.024
	WLLS	3.990	3.429	1.215
	Improved LLS	5.198	4.207	1.480
	Trilateration	6.262	6.282	2.388
$\alpha_{1}=2$, $\sigma_{1}=1$ $\alpha_{2}=1.6$, $\sigma_{2}=6$ $\alpha_{2}=1.6$, $\sigma_{2}=6$	LLS	4.611	3.816	1.790
	WLLS	4.846	3.985	2.034
	Improved LLS	4.793	3.949	1.848
	Trilateration	8.303	13.165	4.999
$\alpha_{1}=2$, $\sigma_{1}=1$ $\alpha_{2}=1.6$, $\sigma_{2}=6$ $\alpha_{3}=1.9$, $\sigma_{3}=6$	LLS	4.991	4.014	1.638
	WLLS	4.518	3.746	2.147
	Improved LLS	5.045	4.086	2.183
	Trilateration	7.852	11.743	4.847
$\alpha_{1}=2$, $\sigma_{1}=1$ $\alpha_{3}=1.9$, $\sigma_{3}=6$ $\alpha_{3}=1.9$, $\sigma_{3}=6$	LLS	4.775	3.869	1.887
	WLLS	5.027	4.074	1.202
	Improved LLS	5.352	4.285	2.806
	Trilateration	7.045	9.625	4.395
$\alpha_{2}=1.6$, $\sigma_{2}=6$ $\alpha_{2}=1.6$, $\sigma_{2}=6$ $\alpha_{3}=1.9$, $\sigma_{3}=6$	LLS	5.041	4.082	1.788
	WLLS	5.017	4.068	1.730
	Improved LLS	4.885	3.928	2.090
	Trilateration	8.582	12.960	5.198
$\alpha_{2}=1.6$, $\sigma_{2}=6$ $\alpha_{3}=1.9$, $\sigma_{3}=6$ $\alpha_{3}=1.9$, $\sigma_{3}=6$	LLS	5.038	4.084	2.023
	WLLS	5.274	4.268	1.754
	Improved LLS	5.291	4.244	2.975
	Trilateration	8.766	11.586	5.024

## Data Availability

Data are contained within the article.
